# The long-term financial consequences of breast cancer: a Danish registry-based cohort study

**DOI:** 10.1186/s12889-017-4839-x

**Published:** 2017-10-30

**Authors:** Laura Schärfe Jensen, Charlotte Overgaard, Henrik Bøggild, Jens Peter Garne, Thomas Lund, Kim Overvad, Kirsten Fonager

**Affiliations:** 1 Department of Social Medicine, Aalborg University Hospital, Havrevangen 1, 2. Sal, 9000 Aalborg, Denmark; 2 Public Health and Epidemiology Group, Department of Health Science and Technology, Aalborg University, Aalborg, Denmark; 3 Department of Epidemiology and Biostatistics, Aalborg University Hospital, Aalborg, Denmark; 4 Department of Breast surgery, Aalborg University Hospital, Aalborg, Denmark; 5 Department of Public Health and Quality Improvement, Central Denmark Region, Aarhus, Denmark; 6 Department of Public Health – Section for Epidemiology, Aarhus University, Aarhus, Denmark; 7 Department of Clinical Medicine, Aalborg University, Aalborg, Denmark

## Abstract

**Background:**

A breast cancer diagnosis affects an individual’s affiliation to labour market, but the long-term consequences of breast cancer on income in a Danish setting have not been examined. The present study investigated whether breast cancer affected future income among Danish women that participated in the work force. We also examined the roles of sociodemographic factors and prior psychiatric medical treatment.

**Methods:**

This registry-based cohort study was based on information retrieved from linked Danish nationwide registries. We compared the incomes of 13,101 women (aged 30–59 years) diagnosed with breast cancer (exposed) to those of 60,819 women without breast cancer (unexposed). Changes in income were examined during a 10-year follow-up; for each follow-up year, we calculated the mean annual income and the relative change compared to the income earned one year prior to diagnosis. Expected changes in Danish female income, according to calendar year and age, were estimated based on information from Statistics Denmark. For exposed and unexposed groups, the observed income changes were dichotomized to those above and those below the expected change in income in the Danish female population. We examined the impact of breast cancer on income each year of follow-up with logistic regression models. Analyses were stratified according to educational level, marital status, and prior psychiatric medical treatment.

**Results:**

Breast cancer had a temporary negative effect on income. The effect was largest during the first three years after diagnosis; thereafter, the gap narrowed between exposed and unexposed cohorts. The odds ratio for an increase in income in the cancer cohort compared to the cancer-free cohort was 0.81 (95% CI 0.77–0.84) after three years. After seven years, no significant difference was observed between cohorts. Stratified analyses demonstrated that the negative effect of breast cancer on income lasted longest among women with high educational levels. Being single or having received psychiatric medical treatment increased the chance to experience an increase in income among women with breast cancer.

**Conclusion:**

A breast cancer diagnosis led to negative effects on income, which ameliorated over the following seven years. Sociodemographic factors and prior psychiatric medical treatment might influence long-term consequences of breast cancer on income.

## Background

After a diagnosis and treatment of breast cancer, the ability to resume employment and a working lifestyle might have both short and long-term impacts on personal financial income. Over the last few years, more attention has been directed towards research on the working lifestyle and employment of women with breast cancer, due to the high incidence of breast cancer [1], the high survival rate [2], and the fact that a large percentage of women are affected at a working age [3].

Previous studies have found a negative association between breast cancer and changes in personal income in the 1–3 years following a diagnosis of breast cancer, in Denmark [4, 5] and other Nordic countries [6–9]. However, study results have been conflicting [10, 11]. The majority of studies has concentrated on the effect of breast cancer on changes in income in the first five years after diagnosis, but negative effects have been demonstrated in 9- and 13-year follow-up data [7, 9]. Previous study findings have been limited by a lack of information regarding income development in the background population, different methods of measuring income and changes in income, and the inclusion of patients over a successive period of years without considering general changes in real income development (real income = income after adjusting for inflation [12]) over the study period. Real income is liable to change over time; for example, it was negative in Denmark in the late 2000’s, due to the Global Financial Crisis, after many years of positive development [13].

In previous studies, the stage of breast cancer was negatively correlated with its impact on income [5, 8, 9]. Additionally, greater impacts were found among women with low levels of education [5, 6, 8, 9] and among older women [8]. Marital status was not reported to affect income development [8].

Depressive symptoms often arise in the wake of breast cancer [14]. A history of major depression prior to a diagnosis of breast cancer was associated with increased risk of experiencing depressive symptoms after the diagnosis [15]. The combination of breast cancer and current or recent depressive symptoms was positively associated with early retirement [16] and unemployment in the years following the diagnosis [17]. Moreover, for most women, changes in income occurred promptly after a breast cancer diagnosis. Mental health problems, per se, are known to hinder participation in the labour market [18, 19], but no study has explored how the combination of a history of psychiatric medical treatment and breast cancer might affect income in the years after a breast cancer diagnosis.

This study aimed to determine whether breast cancer affected changes in future income (we examined up to 10 years of follow-up data) among Danish women that were self-supporting at the time of diagnosis, compared to a cancer-free reference cohort. Furthermore, we aimed to determine whether changes in income differed according to socio-demographic factors and prior psychiatric medical treatment. The changes in income was used in order to vary labour force participation in the years following a diagnosis of breast cancer.

## Methods

We used unique registration numbers (CPR numbers), which are assigned to all Danish citizens, to identify unambiguous linkages between six Danish administrative registries. We defined the exposed group as women diagnosed with breast cancer, and the outcome was the change in income up to one of the following events: death, emigration, early retirement, age pension, or the end of the observation period (31 December 2013). The follow-up period varied between 2 and 10 years.

### Study population

The study included two cohorts: 1) an exposed group of women between 30 and 59 years of age diagnosed with incident breast cancer in 2000–2011; and 2) an unexposed group that comprised women without breast cancer (unexposed); this group included five unexposed subjects age-matched to each patient with breast cancer (exposed); unexposed women had to be alive on the date the matched patient was diagnosed with breast cancer. The unexposed cohort was randomly drawn from the Danish CPR registry. Only women free of breast cancer during the study period were included in the unexposed cohort. Throughout this paper, the term “year of diagnosis” also refers to the year that women were included in the unexposed group.

**Fig. 1:**
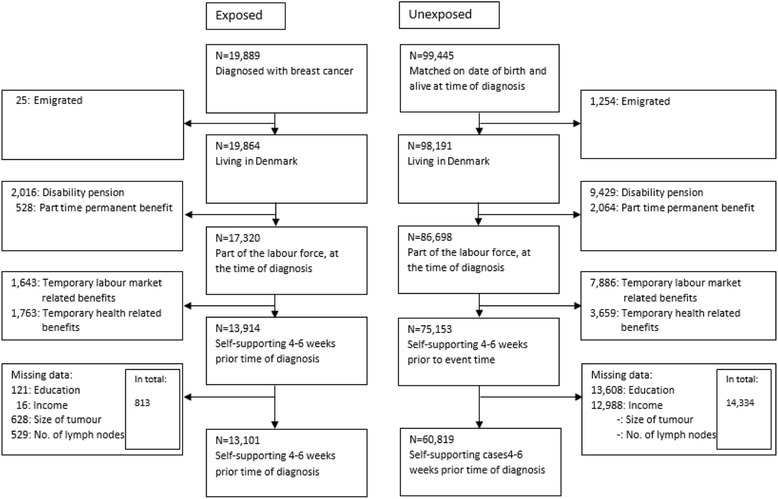
The selection of individuals listed in national databases for inclusion in the final study population

We excluded women that did not reside in Denmark and women that received any kind of permanent or temporary labour market benefits or health-related benefits in the 4- to 6-week period prior to diagnosis. This time window was used for two reasons: 1) no more than 18 days were allowed between a cancer suspicion and the start of treatment according to an agreement between the Danish Government and The Danish Regions, that operates the hospital services, and 2) during the study period, the employer payment period for sick leave compensation varied between 14 and 21 days. Finally, we excluded women when information was missing on income for the year before diagnosis, educational level, tumour size, or the number of affected lymph nodes (Fig. 1, Flowchart).

### Data on exposure

Data on breast cancer was obtained from the Danish Breast Cancer Cooperative Group **(**DBCG), which is a national clinical database for patients with breast cancer in Denmark [20]. Approximately 95% of all Danish women under 75 years of age that are diagnosed with breast cancer are registered in the DBCG [20, 21]. Each record contains histopathological data, information regarding therapeutic interventions, and information regarding prognostic factors. We retrieved data on the year of diagnosis (defined as the date of surgery), clinical characteristics, and treatment modalities. The clinical characteristics included the number of affected lymph nodes (0, 1–3, or >3), and tumour size (≤20, 21–50, or >50 mm). Treatment modalities included the type of surgery (lumpectomy, mastectomy, or biopsy) and adjuvant treatment (i.e., no medical treatment, chemotherapy or endocrine therapy alone, a combination of chemotherapy and endocrine therapy, or not allocated to standard treatment). Radiotherapy was not included among adjuvant treatments due to a large number of missing values (*N* = 1389 ~ 10%).

### Data on demography

Information on demographic characteristics were obtained from the population-based Integrated Database for Labour Market Research (IDA), which has been administered by Statistics Denmark since 1980 [22]. We obtained information about marital status (single or married/cohabiting) and age at the time of diagnosis or matching. Afterwards, age was divided into three different age groups for the following analyses (30–39, 40–49, and 50–59 years old).

### Data on educational status

In Denmark, the Population’s Education Registry holds information on individual education (authorized by the Danish Ministry of Education) after the 8th grade; the records include any education that lasted 80 h or more [23]. From this registry, we retrieved data on the highest educational attainment of individuals in the year of diagnosis. Assessments of education were based on the International Standard Classification of Education (ISCED 2011) [23]. We deviated from the ISCED classification at the fourth level (i.e., post-secondary non-tertiary education), because no programmes of that category exist in Denmark [24]. As a result, we defined three educational levels, as follows: group I, Low levels: early childhood education, primary education, lower and upper secondary education, and high school programmes (ISCED levels 0–3); group II, intermediate levels: short and medium-length higher education and vocational training and education (ISCED levels 5 and 6); and group III: high levels: long-term higher education, bachelor’s, master’s, and PhD degrees (ISCED levels 7 and 8).

### Data on income

Information on annual gross income, both labour and owner income including social benefits and other transfer payments, was extracted from the Personal Income Statistics database. This registry includes information on all Danish residents aged over 14 that are required to pay Danish taxes for the entire year [25]. Income was recorded in units of Danish kroner (DKK), and values were deflated according to the 2009 valuation (the exchange rate was 100$ ≈ 500 DKK). The income one year prior to diagnosis was used as the reference income in the analyses.

### Data on labour market status

The Danish labour market is characterized by a high degree of economic compensation for individuals, in case of unemployment or a reduced ability to work. When individuals are unable to work, due to illness or disability, they can receive sick-leave benefits or apply for a disability pension. Alternatively, when individuals experience a permanent reduction of at least 50% of their working capacity and they have previously exhausted all other avenues of obtaining ordinary employment, they may apply for a part-time permanent benefit. All Danish citizens are eligible for these benefits, irrespective of their job type and insurance status. The Danish DREAM database contains information on all public social benefits paid to Danish citizens [26]. These data are pooled from all relevant Danish ministries, municipalities, and the national bureau of statistics (Statistics Denmark). The DREAM database was proven valid, in terms of public health research [26]. From this registry, we retrieved weekly data for the various types of benefits disbursed to study participants.

Codes from the DREAM registry were used in defining the initial study population. The codes were categorized into six types of non-work related income: *temporary labour-market-related benefits* (unemployment, social assistance, rehabilitation, and vocational rehabilitation benefits); *temporary health-related benefits* (sick-leave benefit); *part-time permanent benefit*; *disability pension*; *voluntary early retirement benefit*; and *age pension*. Two categories described individuals no longer followed in the database: *emigrated* and *dead.*

### Data on psychiatric treatment

The Danish National Prescription Registry [27] holds data for prescriptions filled in the pharmacies of Denmark. The type of medicine is classified by the Anatomical Therapeutic Chemical (ATC) Classification System [28]. From this registry, we retrieved information on *psychiatric medical treatments* (antipsychotics (NO5A), anxiolytics (NO5B), or antidepressants (NO6A)) that had been prescribed to individuals 2, 3, or 4 years prior to the year of diagnosis.

### Statistical analysis

Frequencies and percentages were calculated for all covariates, including age group, educational level, income one year prior to a breast cancer diagnosis, marital status, prior psychiatric medical treatment, year of diagnosis, and clinical variables.

For each year of follow-up, we calculated the mean annual income and percentage-wise changes in income compared to the year before diagnosis for women participating in the work force and for all women. The exposed group was divided into two subgroups according to disease severity: stage IA (tumour size ≤20 mm with no lymph node involvement) and stage IB – stage IV (tumour size >20 mm and/or lymph node involvement) [29]. The classification was used in order to compare the group of women with the best prognosis to the remaining women.

During the study period, the real income changed from positive to negative, as a result of the economic recession [13]. In order to take these changes into account, a calendar year and age specific expected change in real income was estimated, based on information from Statistics Denmark (which holds information about yearly income change in 5 years groups among Danish females). The income change for the exposed and unexposed cohorts were dichotomised, according to whether the change was above or below the expected change in income for the Danish female population. The impact of breast cancer on changes in income for the exposed group compared to the unexposed group for each year of follow-up was estimated by applying logistic regression models; results are expressed as the odds ratio (OR) and 95% confidence interval (CI). In these analyses, only women that participated in the work force at the beginning of the yearly interval were included; thus, we excluded women that died or immigrated together with women that received permanent benefits, early retirement, or an age pension.

We stratified the groups according to educational level, marital status, and prior psychiatric medical treatment in the 2–4 years prior to the diagnosis of breast cancer.

We conducted data management with SAS version 9.4 (Cary), and we performed statistical analyses with STATA version 13 (StataCorp. 2013. *Stata Statistical Software: Release 13*. College Station, TX: StataCorp LP).

### Ethics statement

This study was approved by the Danish Data Protection Agency (Ref. 2008–58-0028). The data for this project are held in the research environment of Statistics Denmark, a state owned institution. It is illegal to export an individual person’s data from this institution and all data are handled with encrypted person identification [30, 31].

## Results

This study included an exposed cohort that comprised 13,104 women diagnosed with breast cancer and an unexposed reference cohort that comprised 60,870 subjects free of breast cancer. Table 1 shows baseline data for the study population, divided into exposed and unexposed groups.

**Table 1:** Baseline characteristics for a cohort of women diagnosed with breast cancer between 2000 and 2011 and a cohort of women without breast cancer (five individuals for each patient with breast cancer, matched for age). All individuals were alive when breast cancer was diagnosed

Characteristics	Women diagnosed with breast cancer total: 13,101 % (N)	Women without breast cancer total: 60,819 % (N)
Sociodemographic factors		
Age at diagnosis (years):		
30-39	8.5 (1,119)	8.5 (5,170)
40-49	33.3 (4,362)	33.2 (20,180)
50-59	58.2 (7,620)	58.3 (35,469)
Highest completed education:		
Low levels (ISCED 0-3)	61.4 (8,047)	63.9 (38,845)
Intermediate levels (ISCED 5-6)	30.6 (4,005)	29.2 (17,767)
High levels (ISCED 7-8)	8.0 (1.054)	6.9 (4,207)
Income (DKK, deflated to 2009):		
First tertile (<267,246) (<53,450$)	30.7 (4,026)	33.5 (20,368)
Second tertile (267,247-348,964) (53,450-69,790$)	32.7 (4,286)	33.1 (20,108)
Third tertile (>348,965) (>69,790$)	36.6 (4,789)	33.5 (20,343)
Marital status:		
Married/cohabiting	58.6 (7,670)	64.6 (39,278)
Single	41.4 (5,431)	35.4 (21,541)
Prior use of psychiatric medical treatment:		
Yes	15.5 (2,028)	14.2 (8,630)
No	84.5 (11,073)	85.8 (52,189)
Clinical factors		
Year of diagnosis:		
2000	7.9 (1,038)	7.9 (4,826)
2001	8.2 (1,080)	8.0 (4,870)
2002	8.7 (1,137)	8.5 (5,137)
2003	8.3 (1,091)	7.9 (4,798)
2004	8.0 (1,053)	8.1 (4,898)
2005	7.5 (985)	7.6 (4,609)
2006	7.1 (934)	7.5 (4,565)
2007	7.9 (1,037)	7.8 (4,718)
2008	8.8 (1,133)	8.8 (5,337)
2009	10.6 (1,384)	10.6 (6,477)
2010	9.3 (1,215)	9.4 (5,700)
2011	7.7 (1,014)	8.0 (4,884)
Type of surgery:		
Lumpectomy	58.6 (7,684)	-
Mastectomy	41.3 (5,407)	-
Biopsy	0.1 (10)	-
Adjuvant treatment:		
No adjuvant treatment	16.0 (2,108)	-
Chemotherapy	18.9 (2,474)	-
Endocrine	13.5 (1,774)	-
Chemotherapy AND endocrine	46.2 (6,049)	-
Not in protocol	5.3 (696)	-
Tumour size (mm):		
1-20	63.2 (8,281)	-
21-50	33.7 (4,419)	-
>50	3.1 (401)	-
Involved lymph nodes:		
0	49.7 (6,505)	-
1-3	34.1 (4,469)	-
≥4	16.2 (2,127)	-

Compared to the unexposed cohort, the exposed cohort included more single women, had higher educational levels, and had a higher mean income prior to diagnosis. In the exposed group, most breast cancers involved a small tumour size and no lymph node involvement; a higher percentage of patients were treated with lumpectomy compared to mastectomy, and nearly half of the treatments included both chemotherapeutics and an endocrine adjuvant treatment.

**Fig. 2:**
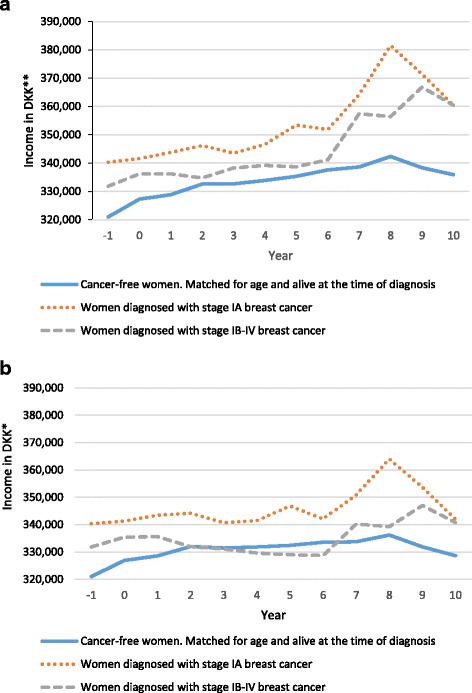
Mean annual income for: **a**) Danish women (aged 30–59) and **b**) Danish women (aged 30–59) that participated in the work force (2000–2011). *100$ ≈ 500 DKK

**Fig. 3:**
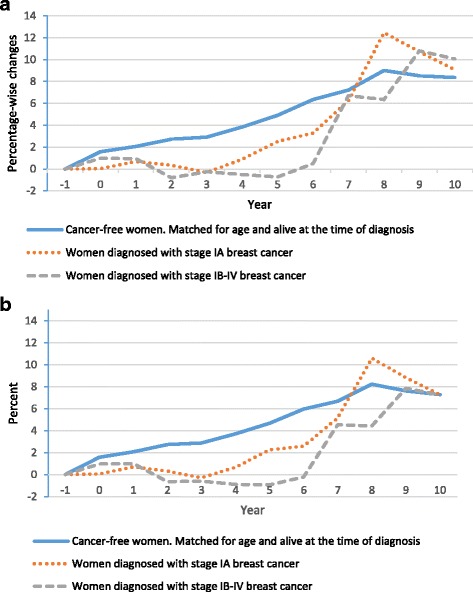
Yearly percent-wise changes in income for: a) Danish women (aged 30–59) and b) Danish women (aged 30–59) that participated in the work force (2000–2011)

Among women that participated in the work force, the unexposed group had a lower mean annual income compared to both of the exposed subgroups (stage IA and stage IB-IV) for all follow-up years (Fig. 2a). The two subgroups of women diagnosed with breast cancer had similar percent changes in income compared to the year prior to diagnosis of breast cancer for the first three years, but the change was not as positive as the change observed in the unexposed group. After seven years, women diagnosed with stage IA cancer experienced the same positive change as women without cancer (7%). After nine years, women diagnosed with stage IB-IV cancers experienced the same positive change as women without cancer (Fig. 2b). Including women receiving permanent benefits in the study population, decreased the annual mean income for the two subgroups of women diagnosed with breast cancer, and caused that women diagnosed with stage IB-IV had a mean annual income below the mean annual income of cancer-free women in year two to six after the year of diagnosis (Fig. 3a). It also decreased the percentage-wise changes in income for both the exposed and the unexposed women, but the pattern of development in percentage-wise changes between the two groups of exposed women and the unexposed women did not change (Fig. 3b).

The following analyses only included women participating in the work force, which means that women who died or emigrated, women who took early retirement, or women receiving age pension, disability pension, or part-time pension were excluded each year of follow-up (Table 2).

**Table 2:** The number of women participating in the work force through all 10 years of follow-up, and the number (and percentages) of women being excluded each year

Exposed women (%)								Unexposed women (%)						
Year	N	D	E	ER	AP	DP	PP	N	D	E	ER	AP	DP	PP
1	13.101	66 (0.5)	8 (0.1)	77 (0.6)		40 (0.3)	52 (0.4)	60,819	56 (0.1)	95 (0.2)	385 (0.6)		88 (0.1)	129 (0.2)
2	12,950	201 (1.6)	13 (0.1)	282 (2.2)		264 (2.1)	356 (2.8)	60,283	89 (0.1)	90 (0.1)	1134 (1.9)		237 (0.4)	259 (0.4)
3	12,454	208 (1.7)	7 (0.1)	307 (2.5)		405 (3.3)	536 (4.3)	58,970	94 (0.2)	62 (0.1)	1338 (2.2)		397 (0.7)	428 (0.7)
4	10,953	236 (2.2)	7 (0.1)	383 (3.5)		480 (4.4)	564 (5.1)	52,720	94 (0.2)	49 (0.1)	1705 (3.3)		598 (1.1)	565 (1.1)
5	9212	169 (1.8)	8 (0.1)	269 (2.9)		509 (5.5)	515 (5.5)	45,460	85 (0.2)	32 (0.1)	1542 (3.4)		690 (1.5)	625 (1.4)
6	7554	131 (1.7)	7 (0.1)	233 (3.1)	52 (0.7)	507 (6.7)	467 (6.2)	37,976	76 (0.2)	40 (0.1)	1407 (3.7)	276 (0.7)	795 (2.1)	620 (1.6)
7	6214	102 (1.6)	5 (0.1)	199 (3.2)	120 (1.9)	446 (7.2)	416 (6.7)	31,527	64 (0.2)	27 (0.1)	1146 (3.6)	537 (1.7)	850 (2.7)	571 (1.8)
8	4984	76 (1.5)	4 (0.1)	148 (3.0)	100 (2.0)	386 (7.7)	365 (7.3)	25,798	61 (0.2)	15 (0.1)	955 (3.7)	432 (1.7)	827 (3.2)	514 (2.0)
9	4010	65 (1.6)	1 (0)	131 (3.2)	92 (2.3)	329 (8.2)	280 (7.0)	20,776	56 (0.3)	16 (0.1)	768 (3.7)	463 (2.2)	717 (3.5)	429 (2.1)
10	3084	35 (1.1)	0 (0)	99 (3.2)	74 (2.4)	263 (8.5)	206 (6.7)	16,187	33 (0.2)	6 (0.1)	622 (3.8)	381 (2.4)	612 (3.8)	339 (2.1)

N = the total number of women used in the analysis each year; D = dead; E = emigrated; ER = early retirement; AP = age pension; DP = disability pension and PP = part-time pension

We used a logistic regression analysis to compare the odds of attaining an increase in income above that expected in the general population of working women (Table 3). There was no significant difference between the two cohorts during the first year. At three years of follow-up, we observed the greatest difference between cohorts (OR = 0.81 (0.77–0.84)). Thereafter, the difference between the two cohorts narrowed, and after seven years, no significant differences were observed.

**Table 3:** The odds (OR, 95% CI) that women diagnosed with breast cancer (exposed) compared to women without breast cancer (unexposed) would experience an increase in income above the increase expected for women in the work force during each year of follow-up

Follow-up	Exposed group		Unexposed group		OR (95% CI)
	N	Proportion with an increase in income^a^ (%)	N	Proportion with an increase in income^a^ (%)	
1 year	12,858	41.1	60,066	42.1	0.96 (0.93–1.00)
2 year	11,834	36.1	58,474	40.4	0.83 (0.80–0.87)
3 year	10,012	33.9	51,895	38.8	0.81 (0.77–0.84)
4 year	8168	33.6	44,297	38.1	0.82 (0.78–0.87)
5 year	6530	32.8	36,661	36.8	0.84 (0.79–0.89)
6 year	5240	31.8	30,112	35.5	0.84 (0.79–0.90)
7 year	4122	33.2	24,377	35.0	0.93 (0.86–0.99)
8 year	3259	33.5	19,435	35.3	0.92 (0.85–1.00)
9 year	2475	33.8	15,041	34.0	0.98 (0.90–1.08)
10 year	1808	34.7	10,999	33.8	1.01 (0.91–1.13)

^a^The increase in income was defined as a larger increase than the change expected, in the general female population, based on age and calendar year

**Fig. 4:**
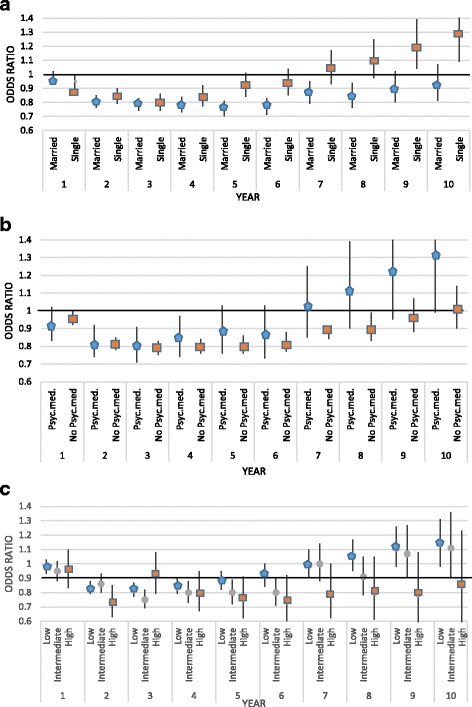
Odds ratios of exposed: unexposed groups for experiencing an increase in income, according to individual characteristics. Groups included Danish women diagnosed with breast cancer (exposed) compared to women without breast (unexposed); the unexposed group was matched to the exposed group for age and alive at the time of breast cancer diagnosis. The odds ratios are stratified according to (**a**) education level (low, intermediate, high), (**b**) marital status (single, married), and (**c**) history of psychiatric medical treatment (yes, no) prior to breast cancer diagnosis

We then stratified the groups according to educational level, marital status, and prior psychiatric medical treatment (Fig. 4) to determine whether any of these factors influenced the impact of breast cancer on income. We found similar patterns in each strata for the first six years; the odds that the exposed groups would experience an increase in income compared to the unexposed groups showed ORs below 1. For the following years, the associations between breast cancer and income changes tended to differ according to marital status and prior psychiatric medical treatment. We found positive associations with a single marital status (Fig. 4a) and prior psychiatric medical treatment (Fig. 4b), although the associations were not statistically significant. For women with high educational levels (Fig. 4c) the impact of breast cancer on income tended to last longer than the impact on women with low educational levels.

## Discussion

This large registry-based cohort study found that a breast cancer diagnosis had a temporary, negative economic significant (with changes above $1000s) impact on changes in personal income over the subsequent 10 years among Danish women that had been self-supporting 4–6 weeks prior to the diagnosis. The temporary negative impact was observed among women diagnosed with both stage IA and stages IB-IV cancers. Regardless of this negative impact, the study also demonstrated that the mean annual income in the exposed cohort exceeded the mean annual income in the unexposed cohort in all follow-up years. This result might be expected, due to the social gradient in breast cancer incidence, because risk is higher among women with high socioeconomic status [32]. The negative impact of breast cancer on yearly percent changes in income was largest after three years. Thereafter, the gap between the exposed and unexposed cohorts narrowed, and after seven years, the yearly percent changes were not significantly different between groups. Nevertheless, the negative impact of breast cancer on changes in income was longer-lasting among women with high educational levels. Moreover, after six years, the association between breast cancer and income changes tended to differ according to marital status and prior psychiatric medical treatment; we observed that, in the exposed group, single women and those that had a history of psychiatric medical treatment were more likely to experience an increase in income, compared to women in the unexposed groups.

### Interpretation

The effect of breast cancer on the yearly percent change in income was larger and longer lasting among women with stages IB-IV compared to women with stage IA breast cancer. This finding was consistent with findings in other Nordic studies [5, 9]. However, we found only a small difference in the impact of breast cancer on income between women diagnosed with breast cancer and women without cancer after one year. In contrast, a previous study [9] found a significant difference in the impact of breast cancer on income between patients with breast cancer and those without breast cancer after one year. This discrepancy may be due to different definitions of income. In the present study, we included social benefits (e.g., sick-leave benefits and unemployment benefits) in the income variable; in contrast, the other study defined income as solely earnings from employment.

Changes in real income over time can be caused by inflation and by nationwide and worldwide events; e.g., legislative changes related to sick leave benefits [33] and the Global Financial Crisis [13]. These changes make comparisons over time difficult. Inflation can be taken into account by deflating the income to the value of currency in a certain year; some existing studies have applied deflation to their analyses [5, 7, 9]. However, the impact of events might be more difficult to address. In Denmark, the real income for women 30–60 years old increased from 2000 to 2009, and then decreased in 2010–2011, due to the Global Financial Crisis [13]. The present study took this variability into account by estimating age- and calendar year-specific expected changes; then, we used those values as thresholds for categorizing the yearly changes in income as increasing or decreasing. Nevertheless, our results were consistent with most other studies, regardless of whether they performed this adjustment [5–9, 11].

In this study, the negative impact of breast cancer on changes in income tended to be longer lasting among women with high educational levels, compared to women with low educational levels. Another Nordic study reported that the impact of breast cancer was more pronounced among women with low educational levels compared to women with high-educational levels [8]. However, Syse et al. followed women for 1 to 8 years, and the impacts on income in different educational subgroups were analysed based on the impact averaged over all follow-up years, while this study analysed yearly impacts demonstrating no clear difference between educational subgroups during the first six years after diagnosis.

In the exposed group, marriage reduced the likelihood that women might experience an increase in income compared to women in the unexposed group. This observation might reflect the possibility that married women could be supported by their spouses, and therefore, these women would experience a decrease in income. In contrast, Syse et al. [8] found no difference in income changes between subgroups based on marital status. However, the cohort studied by Syse et al. had different types of cancer (only 44% had breast cancer), which were associated with different prognoses, and thus, they had different effects on income. Furthermore, the conflicting results between studies might be due to different legislation in the two countries or the different age-groups examined (ages 30–59 years in the present study, versus ages 40–59 years in the study by Syse et al.). Indeed, compared to older women, younger women are often diagnosed with more aggressive breast cancers [34], which lead to more extensive treatment, more side-effects (both short- and long term) [35], and an increased risk of local recurrence [36]. All these factors are likely to hinder future participation in the labour market.

No significant differences were found between women with and women without a history of psychiatric medical treatment prior to their diagnosis of breast cancer. This finding might indicate that the women that managed to support themselves, despite prior psychiatric medical treatment, could also manage further psychological strains, like being diagnosed and treated for breast cancer. However, because we included only women that were self-supporting before the breast cancer diagnosis, we may have excluded women with more severe psychiatric disorders.

### Strengths and limitations

This study had several strengths, including the large sample size, the long follow-up period, and the use of unique Danish registries that contained updated information on, e.g., labour market status and income. The DBCG has demonstrated high data completeness with good data validity [20]. This feature was also shown for the other registries used in this study [27, 37].

Because of the long study period and long follow-up period of this study, we created a variable that captured age- and calendar year-specific changes in average incomes for the general Danish female population. Thus, we could determine whether the yearly change in income for an individual was higher or lower than the income change expected in the general population. This variable was intended to provide adjustments for both nationwide and international events that could cause general income changes, and thus, including this variable improved the accuracy of comparisons over time.

This study also had some limitations. The long follow-up period increased the probability that only women with a strong affiliation to the labour market were included. The women that survived and participated in the work force for ten years after diagnosis were younger and they were diagnosed in the beginning of the study period. This means, that they might have received less comprehensive treatment than women diagnosed at later times due to changes in treatment protocols during the study period. The more comprehensive treatment increase the survival rate, but it also increase the risk for long-term side effects from treatment, which might hinder participating in the labour market. Furthermore, legislative changes in relation to sick leave and changes in the world economic during those years might also have distorted the results. The last-mentioned limitation has been taken into account using the age and year specific variable. We cannot rule out the possibility that the long-term consequences of breast cancer might be different among women diagnosed late in the study period.

Also, by including only women that were self-supporting in the 4 to 6 weeks prior to the breast cancer diagnosis, we may have excluded some women that received neo-adjuvant treatment prior to surgery (only a small percentage was included in the present study). Women that receive neo-adjuvant treatment tend to have more serious prognoses; thus, in excluding that portion of the population, we might have underestimated the effect of breast cancer on income. However, we suspect that this factor probably did not have a large influence on our results, because we only included women that were participating in the work force.

Another limitation of the study was our use of psychiatric medical treatment as a measure for mental health problems. In addition to medications, psychiatric treatment modalities consist of consultations with psychologists or psychiatrists in private practice or in the hospital and admissions to psychiatric hospitals. There are two primary limitations in using psychiatric medical treatment as a measure of mental health problems. First, although psychiatric medications are prescribed for the majority of patients with psychiatric disorders, some patients do not receive prescriptions for medications. Individuals with mild and moderate depressive symptoms may be treated only with psychotherapy [38]; thus, in the present study, patients with psychiatric problems that did not receive medications would not be classified as individuals with a history of mental health problems. The second problem was that psychiatric medical treatments are sometimes prescribed for other diagnoses, like neuropathic pain [39]; thus, these women might be misclassified as individuals with a history of mental health problems. Any misclassification due to these limitations could lead to an underestimation of differences between subgroups of patients with and without a history of psychiatric medical treatment.

The results of this study may not be generalizable to other countries, due to differences in legislation related to sick-leave and differences in benefits awarded by the welfare system. Furthermore, the results may not be generalizable to other types of cancer, due to the relatively good prognosis of breast cancer and the fact that a high percentage of women are able to resume work in the years following a breast cancer diagnosis [40, 41]. Finally, examining income changes only among women that participated in the work force made it difficult to generalize our results to all patients with breast cancer because the general population of patients with breast cancer has increased rates of receiving e.g. disability pensions and taking early retirement. Indeed, it could be argued that the results of the present study have underestimated the economic consequences of breast cancer, because during follow up, a large number of patients received permanent benefits, and therefore, were excluded from our analyses. Including women receiving permanent benefits in the descriptive analyses increased the decrease in income and thereby verified the suggested underestimation of the economic consequences of breast cancer. However, the present study aimed to examine the consequences specifically for women that could participate in the work force. Previous studies have demonstrated, that a diagnosis of breast cancer negatively impacted income, due to the increased risks of receiving benefits through unemployment [17], early retirement [16], and disability pensions [42, 43]. The present study demonstrated, that not all changes in income can be explained by the increased risk of receiving permanent benefits. Other explanations might be e.g. changes in priorities [44, 45]or employer discrimination [46].

## Conclusion

This study focused on the impact of a breast cancer diagnosis on income among Danish women, aged 30–59 years, which were self-supporting prior to the diagnosis of breast cancer, compared to women without breast cancer. We found that the diagnosis and treatment of breast cancer caused a significant decrease in income for the following seven years. The biggest difference in income change was observed after three years. We also found that, for the first six years after diagnosis, the effects on income were similar for individuals with different educational levels, marital statuses, and histories of psychiatric medical treatment. However, after six years, our results demonstrated that the impact on income was more long lasting among women with high educational levels; moreover, the long-term impact was positively associated with a single marital status and with a history of psychiatric medical treatment.
